# Small animal PET based on 16x16 TSV-MPPCs and monolithic crystals

**DOI:** 10.1186/2197-7364-2-S1-A16

**Published:** 2015-05-18

**Authors:** Antonio Gonzalez, Albert Talens Aguilar, Pablo Conde, Liczandro Hernadez Hernadez, Luis Fernando Vidal San Sebastian, Carlos Correcher Salbador, Cesar Molinos Solsona, Sven Junge, Konrad Lankes, Jose Maria Benlloch

**Affiliations:** Institute for Instrumentation in Molecular Imaging, i3M-CSIC, Valencia, Spain; Oncovision, Valencia, Spain; Bruker BioSpin, Germany

In this work we present the design of a small animal PET based on 8 high-density arrays of MPPCs and monolithic scintillators. The MPPCs arrays are composed of 16x16 TSV-type (3x3 mm^2^) elements covering a rough active area of 5x5 cm^2^. A single LYSO block with a thickness of 10mm has been mounted on each detector. Black paint has been applied to the entrance and lateral faces of the crystal to preserve the scintillation light distribution. The axial and transaxial FOVs of one ring are 48 mm and 80 mm, respectively. Each MPPC array has been directly attached to a resistive readout circuit that provides outputs for each row and column of the array. These 32 signals are read with flexible boards 30 cm apart from the PET detector without any additional connectors in between. The PET-system is intended for in-line acquisition in front of MR scanners and as PET-insert inside the sensitive MRI volume. For this purpose, it is necessary to avoid magnetic sensible materials, such as nickel, and to prevent eddy currents in metallic structures induced by the MRI gradients. All detectors are air cooled and kept at temperatures of approximately 20^o^C with a variation below 0.05^o^C. The intrinsic resolution is 2.2 mm at the crystal center (averaged over all 2.6 mm) when Center of Gravity methods are used to resolve the impact position. This value is about a factor 1.5 better than results obtained with the H8500 PSPMT (64 PADs) and similar scintillators. With an improved collimator with holes with only 0.8mm diameter and a length of 70 mm, an intrinsic detector resolution of 1.1mm was reached. The energy resolutions of ROIs of 1x1 cm^2^ showed FWHM values in the range of 14%.

